# Transactions of the Medical Association, New York

**Published:** 1853-10

**Authors:** 


### TRANSACTIONS OF THE MEDICAL ASSOCIATION, NEW YORK.

The Medical Association of Southern Central New York held their seventh annual meeting in June last, and the fruits are a neat pamphlet of 96 pages, containing reports on a variety of surgical and medical subjects. This Association manifests a commendable spirit of industry and perseverance. The members appear to be working men. A number of the reports before us are elaborate and full. There are surgical reports on the counties of Cortland, Broome, Tioga, and Tompkins, that contain valuable facts and statistics; and the report on the endemics and epidemics of Chemung county, though short, is interesting. In a brief notice of epidemic dysentery Dr. BURDICK speaks of a combination of opium and acetate of lead as having been entirely successful in his hands. Dr. BRIGGS has an excellent essay on the diseases incident to adventurers in California; and there is also a good account of hay asthma by Dr. Wey. As a prophylactic, he recommends before the attack is expected, the cold shower bath, with quinine and sulphate of iron. Dr. Elliottson recommends persons subject to this affection to sleep with chloride of lime in their rooms about the period of the anticipated attack.

